# One Catheter, Two Coronaries. Haven’t We Seen This Before?

**DOI:** 10.36660/abc.20190684

**Published:** 2019-11

**Authors:** Cesar Rocha Medeiros

**Affiliations:** 1 Instituto Nacional de Cardiologia, Rio de Janeiro, RJ - Brazil; 2 Hospital Badim, Rio de Janeiro, RJ - Brazil; 3 Hospital Unimed, Rio de Janeiro, RJ - Brazil

**Keywords:** Coronary Angiography, Coronary Artery Disease, Cardiac Catheterization/methods, Cardiac Catheters, Radiation Dosage.

Radial access is the default for diagnostic coronariography in many centers and, according to the latest consensus, should be in all.^1^

Over these more than 60 years, coronariography has been performed by several techniques. One of them, ironically the very first one, using just one catheter to canulate both coronaries and enter the left ventricle.

With the advent of thinner catheters, better images and less toxic dye media, we’ve moved towards safest, fastest and less invasive procedures.

The catheter chosen for this study’s particular comparison is one of many suitable to cannulate both coronaries when friendly anatomy is present.^2^ The list includes Multipurpose, Amplatz left, Sones type II, etc. These catheters are able to, in a majority of patients, engage the coronaries in a coaxial way, allowing a good quality angiography.

In the current article, authors compare one particular catheter shape (Tiger 1), with the standard catheters dedicated to engage each coronary (Judkins right and left), originally made for femoral approach, but widely adapted for radial access.

The primary goal was to show that, using a single catheter, operators would be able to reduce contrast media amount; reducing also procedural time, radiation, patient discomfort and costs.

The authors were able to show that, using a single catheter, less contrast was necessary, the fluoroscopy time was shorter, less spasm was noticed and the procedure costs were lowered.

A single catheter approach has the obvious advantage of less catheter trade and, consequently, less manipulation of the arterial path, which can account for less spasm and more comfort for the patient. Procedural time is also expected to reduce, and this was shown in the article indirectly, utilizing fluoroscopy time.

These hypotheses were tested and proved before,^3,4^ but the current article brings us one extra precious information since it precifies the procedure in our country’s environment and shows that a single-catheter approach reduces costs when compared to two catheters. The impact of reducing costs in a poor country, where the public health system has serious issues is paramount and should be incentivated. For this reason, the findings of this study have to be published and tried to be reproduced in a larger scale.

It is important to say that these data derive from retrospective analysis done in a single center with experienced radial operators. And even in this most selected scenario, a single-catheter approach was utilized in less than 15% of procedures. The extrapolation of this information should be done cautiously, before recomending a single-catheter approach for every radial coronariography.

But even so, Dr Mason Sones keeps leading the way, 60 years after.
